# Agonist-induced activation of human FFA1 receptor signals to extracellular signal-regulated kinase 1 and 2 through Gq- and Gi-coupled signaling cascades

**DOI:** 10.1186/s11658-017-0043-3

**Published:** 2017-07-21

**Authors:** Jing Qian, Yuyang Gu, Chun Wu, Feng Yu, Yuqi Chen, Jingmei Zhu, Xingyi Yao, Chen Bei, Qingqing Zhu

**Affiliations:** 1 Huzhou University Schools of Nursing and Medicine, Huzhou University, HuZhou, 313000 China; 2 Institute of Biochemistry, College of Life Science, Zijingang Campus, Zhejiang University, Hangzhou, Zhejiang 310058 China

**Keywords:** FFA1, Phosphatidylinositol-specific phospholipase C, Extracellular signal-regulated kinase 1 and 2, Gαq/11, Gαi/o

## Abstract

**Background:**

FFA1 is abundantly expressed in the liver, skeletal muscle, monocytes and nervous system, but is particularly abundant in pancreatic β cells. It is widely believed that FFA1 exerts its regulatory roles in a variety of physiological and pathological functions. In response to oleic acid, FFA1 has been shown to induce the activation of extracellular signal-regulated kinase 1 and 2 (ERK1/2) through a mechanism involving EGFR transactivation in a breast cancer cell line. However, the underlying molecular mechanism for ERK1/2 activation mediated by n-6 free fatty acid (LA) in HEK293 cells remains to be further elucidated.

**Methods:**

A FLAG-FFA1 vector was stably expressed in HEK293 cells. Western blot analysis was applied to investigate the change in LA-induced ERK1/2 phosphorylation change in response to kinase inhibitors. Arrestin-2/3-specific siRNA was used to analyze the effect of arrestin-2/3 knockdown on FFA1-mediated ERK1/2 activation.

**Results:**

We proved that activation of ERK1/2 by LA was rapid, peaking at 5 min. Further experiments proved that FFA1 couples to a Gq protein and activates PI-PLC, which induces the IP3/Ca^2+^ and DAG/PKC signal pathways, both of which are involved in ERK1/2 activation. We also showed that there is no EGFR transactivation, arrestin-2/3 or Gβγ pathway participation in ERK1/2 phosphorylation. Treating cells with PTX abolished ERK1/2 activation at a late time point (≥20 min), indicating a critical role for Gi subunits in FFA1-mediated ERK1/2 activation.

**Conclusions:**

Our study provides a detailed delineation of the LA-mediated activation of ERK1/2 in HEK293 cells that are stably transfected with human FFA1. We also present evidence of Gi/Gq-induced synergism in the regulation of ERK1/2 phosphorylation. These observations may provide new insights into the pharmacological effects of FFA1 and the physiological functions modulated by FFA1-mediated activation of ERK1/2.

**Electronic supplementary material:**

The online version of this article (doi:10.1186/s11658-017-0043-3) contains supplementary material, which is available to authorized users.

## Background

FFA1 was sequenced and identified as a member of a subfamily of intronless GPCRs. The family includes GPR41, GPR42 and GPR43, all of which reside on chromosome 19q13.1. The receptor encoded by FFA1 contains two N glycosylation sites (N-X-S/T), five protein kinase C (PKC) phosphorylation sites, and a cysteine at the C-terminus [1]. Both saturated and unsaturated medium- and long-chain (C12-C22) free fatty acids (FFA) were identified as endogenous ligands for FFA1 [2]. FFA1 is abundantly expressed in rodent insulin-secreting cell lines, including INS-1E cells [3], Min6 cells [4] and pancreatic β cells [2]. It is also found in the human brain [5]. Accumulating evidence shows that FFA1 plays a crucial role in the regulation of glucose homeostasis mediated by free fatty acid-induced insulin secretion [6, 7]. In addition to the regulation of endocrine function, FFA1 is involved in bone remodeling, inflammation and neurogenesis [8–10]. These findings suggest that FFA1 may work through multiple pathways in the regulation of different physiological functions.

The basic understanding of G-protein-coupled receptors (GPCRs) is that almost all signal through extracellular signal-regulated kinase (ERK) signaling cascades, which are associated with about 200 cellular substrates and mediate a variety of cellular processes, including proliferation, differentiation, migration, survival and apoptosis [11–13].

FFA1 is activated to elicit an increase in intracellular Ca^2+^ levels via the Gq-dependent pathway, leading to enhancement of glucose-stimulated insulin secretion [14, 15]. In addition, FFA1 has been shown to induce ERK1/2 activation through a mechanism involving Src kinase and EGFR transactivation by oleic acid in the breast cancer cell lines MCF-7 and MDA-MB-231 [16]. By contrast, another study revealed that FFA1 activation by n-3 fatty acids can abolish EGF-induced proliferation and migration in MCF-7 and MDA-MB-231 cells [17]. This suggests that the effects of different FFA1 agonists are different.

In mouse embryonic stem cells, LA regulates various cell cycle proteins via p44/42 MAPK signaling [18]. Interestingly, unsaturated fatty acids promote the activation of ERK1/2 mainly via FFA1, leading to an anti-lipoapoptotic effect on NIT-1 cells [19]. Thus, the underlying mechanism regulating ERK1/2 activation mediated by the n-6 free fatty acids (LA) in HEK293 cells through FFA1 remains unclear.

It is now known that GPCRs regulate MAPK cascades via distinct Gi-, Gs-, Gq/11- and Gβγ- dependent signaling pathways, leading to activation of ERK1/2 [20]. G proteins have complex and diverse roles in the FFA1 signal pathway. Pharmacological inhibitionof Gq/11 blocked Ca^2+^ release from the ER in the β cell line INS-1E [3]. In HEK cells, TUG424-mediating FFA1 downstream signaling is inhibited by pertussis toxin (PTX), which indicates that Gi/o is partially involved [21]. Moreover, LA have been proven to decrease the voltage-gated K^+^ current through FFA1/Gs/cAMP/protein kinase A (PKA) [22]. Further exploration of G protein subunits in FFA1-mediated ERK1/2 activation will be important to better understand the role of FFA1 in various physiological functions.

## Methods

### Materials

Lipofectamine 2000, G418 and Opti-MEM were purchased from Invitrogen. Cell culture media and fetal bovine serum were obtained from Hyclone. Pertussis toxin (PTX), Go6983 and thapsigargin were purchased from Sigma. UBO-QIC was purchased from Dr. E. Kostenis at the University of Bonn. U0126, tyrphostin AG1478, GM6001, PP2 and ET-18-och3 were from Calbiochem. Monoclonal anti-FLAG antibody was purchased from BD Biosciences Pharmingen. Anti-phospho-ERK1/2 (Thr-202/Tyr-204) and ERK1/2 antibodies and horseradish peroxidase-conjugated anti-rabbit IgG were from Cell Signaling Technology. Anti-tubulin antibody was from Beyotime. The β-adrenergic receptor kinase COOH domain (495-689aa), adrenergic and the Gα subunit of transducin plasmid were from the lab of Dr. Naiming Zhou in Zhejiang University.

### Cell culture

FFA1 (GenBank accession no.NM_005303.2) was cloned via PCR using human genomic DNA as a template, with the primers:

5′-AAGCTTGCCACCATGGACCTGCCCCCGCAGCTCTCC-3′ (forward) and.

5′-GGTACCGTCTTCTGGGACTTGCCCCCTTGCGT-3′ (reverse).

The PCR products were inserted into the *HindIII* and *Bgl* sites of the pCMV-Flag vector. HEK293 cells stably expressing human FFA1 were grown in DMEM supplemented with 10% (*v*/v) fetal bovine serum and 800 mg/l G418. Plasmid constructs were transfected into HEK293 cells using Lipofectamine 2000 according to the manufacturer’s instructions. All cells were incubated at 37 °C in a humidified atmosphere of 5% CO_2_, 95% air.

### Small interfering RNAs and siRNA Transfection

Small interfering RNAs (siRNAs) for arrestin-2 and arrestin-3 were purchased as a SMART pool from Dharmacon RNA Technologies. The clathrin HC siRNA was from Santa Cruz Biotechnology. A nonspecific RNA was used as the control for all siRNA experiments. The arrestin and CHC siRNAs were transfected according to the manufacturer’s instructions. Briefly, the first siRNA transfection was performed using Lipofectamine 2000 and Opti-MEM from Invitrogen. After 6–8 h, the cells were divided between new 6-cm dishes. On day 2, a second siRNA transfection was performed. After 24 h, transfected cells were divided for use in various assays.

### Western blot analysis

Cells were plated on 6-well plates, grown to 80% confluence, rinsed with serum-free DMEM, and incubated overnight in serum-free medium. For PTX treatment, the cells were pretreated with 100 ng/ml PTX overnight prior to the ERK1/2 assay. Cells were pre-incubated with various inhibitors for 1 h before activation with the indicated ligands. Ligand incubation was ended by washing the cells with 2 ml of ice-cold PBS followed by the addition of the lysis buffer (1% Triton X-100, 1% deoxycholate and 0.1% SDS) at 4 °C on a rocker for 30 min. The lysates were centrifuged at 4 °C at 10000 g for 15 min. About 40 μg protein underwent electrophoresis on a 10% SDS polyacrylamide gel, then was transferred to a PVDF membrane and immunoblotted using monoclonal anti-pERK1/2 antibody from Cell Signaling Technology. Blots were probed with horseradish peroxidase-labeled secondary antibodies, and chemiluminescence was produced using HRP substrate (Cell Signaling Technology). The working HRP substrate was prepared by combining equal volumes of luminol reagent and peroxide solution. The HRP substrate produces a high intensity signal with low background for detection of both high and low abundance proteins. The blots were stripped and reprobed using an anti-total ERK1/2 (1:2000) monoclonal antibody as a control for protein loading.

To analyze the knockdown of siRNA-targeted proteins, siRNA-transfected Flag-FFA1-HEK293 cells were seeded in a 6-well plate and treated as described above. After blocking, the PVDF membranes were incubated with anti-arrestins (1/1000) and probed with horseradish peroxidase-labeled secondary antibodies. Chemiluminescence was detected using an HRP substrate (Cell Signaling Technology). The blots were stripped and reprobed using an anti-total ERK1/2 (1:2000) monoclonal antibody as a control for protein loading, and all the immunoblots were visualized and quantified using the Bio-Rad Quantity One imaging system.

### Cell viability assay

Cell viability was evaluated using CCK8 assay reagent (Beyotime Biotechnology). The FFA1-HEK293 cells were plated on 96-well plates. After pretreatment with PTX (100 ng/ml) for the indicated time, the viability of the FFA1-HEK293 cells was assessed using CCK8 to detect the soluble orange formazan generated by mitochondrial dehydrogenases, according to the manufacturer’s instructions. The colorimetric signal of each sample was detected and analyzed using a FLUOstar OPTIMA microplate reader (BMG LABTECH Inc.).

### Data analysis

All results are expressed as means ± S.E. of N. Statistical significance was determined using Student’s t-test. Probability values less than or equal to 0.05 were considered significant.

## Results

### Involvement of Gq/PI-PLC downstream signaling in ERK1/2 Phosphorylation

**Fig. 1:**
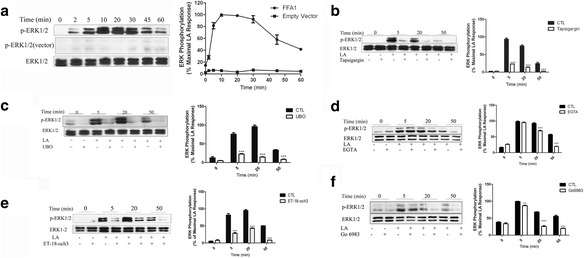
Involvement of IP3/Ca^2+^ and DAG/PKC in FFA1-mediated ERK1/2 activation. **a** – HEK293 cells expressing FLAG-FFA1 or an empty vector(pCMV-Flag) were starved in serum-free media for 18–24 h followed by a challenge with 10 μM LA for the indicated time periods. ERK1/2 phosphorylation was assessed using western blot as described in the Methods section, and corresponding immunoblots were quantified using the Bio-Rad Quantity One Imaging system. **b** – Serum-starved FFA1-HEK293 cells were pretreated with DMSO or thapsigargin (0.5 μM) for 1 h, and the cells were then stimulated with 10 μM LA for the indicated time. **c** – Serum-starved FFA1-HEK293 cells were pretreated with DMSO or UBO-QIC (1 μM) for 1 h, and the cells were then stimulated with 10 μM LA for the indicated time. **d** – Serum-starved FFA1-HEK293 cells were pretreated with DMSO or calcium chelator EGTA (10 μM) for 1 h, and the cells were then stimulated with 10 μM LA for the indicated time. **e** – Serum-starved FFA1-HEK293 cells were pretreated with DMSO or phosphoinositide-specific phospholipase C (PI-PLC) inhibitor ET-18-och-3 (10 μM) for 1 h, and the cells were stimulated with 10 μM LA for the indicated time. **f** – To investigate the role of PKC, serum-starved FFA1-HEK293 cells were pretreated with DMSO or Go6983 (10 μM) for 1 h, and then stimulated with 10 μM LA for the indicated time. Error bars represent the SEM for three replicates. The data shown are representative of at least three replicate independent experiments. Data were analyzed using Student’s t-test. **p < 0.001; ***p < 0.001

LA is known to be an endogenic ligand for FFA1. An expression vector containing human FFA1 fused with Flag-tag was constructed and stably expressed in HEK293 cells. Our recent studies demonstrated that the LA-induced internalization of FFA1 is regulated by G-protein-coupled receptor kinase 2 and arrestin-3. In addition, LA induced a concentration-dependent activation of ERK1/2 with an EC_50_ of 8.669 μM in the stably transfected cells [23]. As illustrated in Fig. 1a, FFA1-initiated activation of ERK1/2 occurred in a time-dependent manner with a maximal activation at 10 min and with a subsequent reduction to base line by 60 min after stimulation with LA. Fasiglifam (TAK-875) enhances the glucose-stimulated insulin secretion (GSIS) pathway via IP3-induced Ca^2+^ oscillations and the DAG/protein kinase C-independent (PKC-independent) Ca^2+^ oscillations mechanism [24]. We speculated that Gq plays a crucial role in the process of FFA1 downstream ERK1/2 activation.

To investigate the role of Gq protein in FFA1-mediated activation of ERK1/2, cells were cultured in the presence or absence of UBO-QIC in DMEM. As shown in Fig. 1c, pretreatment with UBO-QIC resulted in nearly complete inhibition of ERK1/2. Pretreatment of FFA1-HEK293 cells with the PKC kinase inhibitor Go6983 for the time course shown in Fig. 1f, resulted in a significantly decrease in ERK1/2 at early (≤ 5 min) and late time points (≥ 5 min).

To assess whether IP3/Ca^2+^ mobilization was involved in the LA-induced ERK1/2 signal pathway, cells were pre-incubated with the calcium chelator EGTA and endoplasmic reticulum Ca^2+^ ATPase inhibitor thapsigargin. LA-stimulated ERK1/2 phosphorylation was significantly inhibited by EGTA and thapsigargin at late time points (≥ 20 min; Fig. 1b and d).

To confirm that IP3-mediated calcium mobilization increases ERK1/2 phosphorylation, FFA1-HEK293 cells were pretreated with the phosphatidylinositol phospholipase C (PI-PLC) inhibitor edelfosine (ET-18-och3) for 1 h, followed by stimulation with LA for different lengths of time. Upon stimulation with LA, a significant inhibition of FFA1-mediated ERK1/2 activation was observed (Fig. 1e). Collectively, these data demonstrate that FFA1 mediates ERK1/2 signaling phosphorylation via dual mechanisms in which Gq/PI-PLC activates IP3/Ca^2+^ and the DAG/PKC signaling pathway.

### FFA1-mediated ERK1/2 Phosphorylation is not dependent on a Transactivation mechanism involving growth factor receptor

It is generally accepted that the transactivation of growth factor receptors participates in GPCR-mediated ERK1/2 phosphorylation [25]. Breast cancer cells are known to transactivate ERK1/2 through FFA1, involving Src and EGFR [16]. To assess the role of EGFR in the FFA1-induced ERK1/2 pathway in HEK293 cells, serum-starved FFA1-HEK293 cells were treated with AG1478 (EGF receptor inhibitor) and GM6001 (MMP inhibitor), followed by stimulation with LA.

**Fig. 2:**
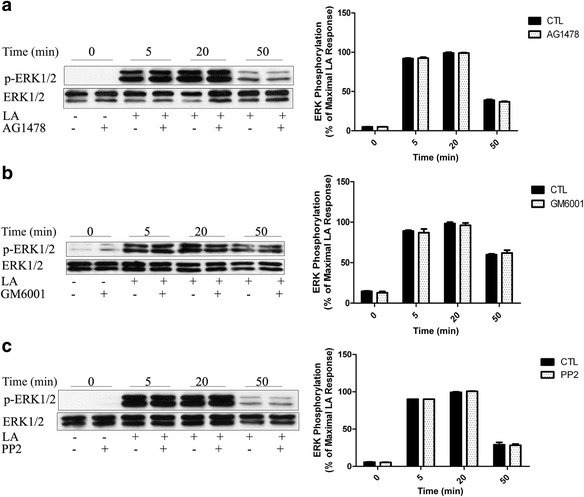
FFA1-mediated ERK1/2 phosphorylation is not dependent on growth factor receptor-involved transactivation. Serum-starved FFA1-HEK293 cells were pretreated with DMSO and (**a**) EGFR-selective receptor tyrosine kinase inhibitor typhostin AG1478 (100 nM), (**b**) MMP inhibitor GM6001 (10 μM), or (**c**) Src-family tyrosine kinase inhibitor PP2 (10 μM) for 1 h. The cells were then stimulated with 10 μM LA for the indicated time. The data shown are representative of at least three replicate independent experiments. Error bars represent the SEM for three replicates. Data were analyzed using Student’s t-test

As shown in Figs. 2a and b, inhibition of EGFR and MMP had no effect on ERK1/2 activation in response to LA in HEK293 cells stably expressing FFA1. Similar results were observed in FFA1-mediated ERK1/2 phosphorylation pre-incubated with the selective Src kinase inhibitor PP2 (Fig. 2c). It is indicated that the non-receptor receptor tyrosine kinase (RTK) Src is not required for FFA1-mediated ERK1/2 activation.

### Gq and Gi protein is involved in ERK1/2 activation without involvement of Gβγ and Arrestins

The Gβγ subunit of heterotrimeric G proteins has been demonstrated to participate in Gi- and Gq/11-coupled receptor ERK1/2 activation [26]. In this study, we revealed that, upon activation of FFA1 by LA, Gq/11 plays a central role in ERK1/2 activation.

**Fig. 3:**
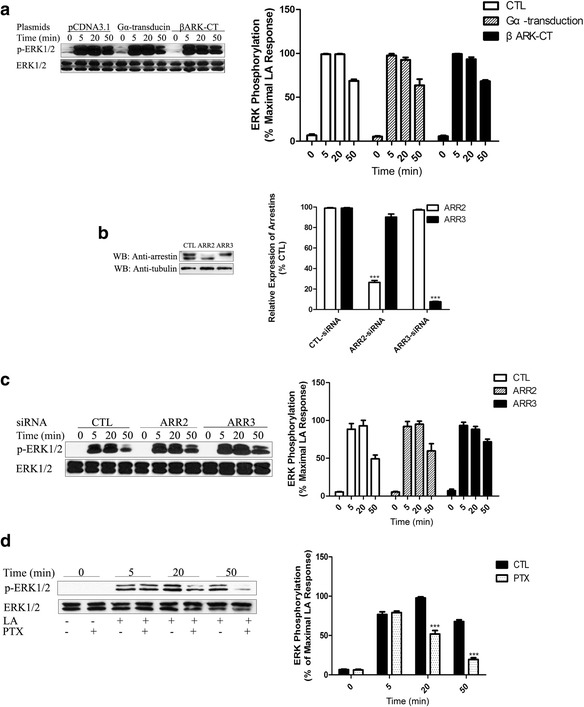
Gi protein is involved in ERK1/2 activation, but Gβγ and arrestins are not. **a** – FFA1-HEK293 cells were transiently transfected with pCDNA3.1 vector or β-adrenergic receptor kinase COOH domain (495-689aa; βARK-CT) or the Gα subunit of transducin. Both βARK-CT and Gα transducin are scavengers of G_βγ_-subunits. The cells were then serum-starved for 24 h and stimulated with 10 μM LA for the indicated periods. **b** – FFA1-HEK293 cells were transfected with specific arrestin siRNAs or a nonspecific control siRNA. 72 h after transfection, the cells were harvested and equal amounts of total cellular lysate were separated by 10% SDS-PAGE, transferred to a PDVF membrane, and incubated with anti-arrestin antibody. Blots were stripped and reprobed for α-tubulin to control for loading. **c** – After transfection with specific arrestin siRNAs or nonspecific control siRNA, cells were stimulated with 10 μM LA for the indicated times and immunoblotted using monoclonal anti-phospho-MAPK E10 (Thr-202/Tyr-204). The bolts were stripped and reprobed for total ERK to control for loading. **d** – FFA1-HEK293 cells were cultured in serum-free DMEM with or without 100 ng/ml PTX for 12 h. The next day, the medium was removed and fresh serum-free DMEM with or without PTX was added for 1 h, and the cells were then stimulated with LA for the indicated times. The data shown are representative of at least three replicate independent experiments. Error bars represent the SEM for three replicates. Data were analyzed using Student’s t-test. ***p < 0.001

Next, we sought to define the role of the Gβγ subunit in FFA1-induced ERK1/2 phosphorylation. The β-adrenergic receptor kinase COOH domain (amino acids 495–689; βARK1-CT) and the Gα subunit of transducin, both of which are scavengers of the Gβγ-subunits, were transfected into FFA1-HEK293 cells. However, there is no inhibition of the LA-mediated 2 increase/2 increase (Fig. 3a). Recent studies have indicated that β-arrestins function as signal transducers for many GPCRs to mediate ERK1/2 activation [27]. However, the result obtained from siRNA knockdown of arrestins exhibited no inhibition of FFA1-induced ERK1/2 phosphorylation (Figs. 3b and c).

To further confirm the role of Gi in LA-triggered ERK1/2 activation, FFA1-HEK293 cells were cultured in the presence or absence of 100 ng/ml of the Gi protein inhibitor PTX, which prevents the Gi protein from interacting with GPCRs at the cell membrane. Treatment of FFA1-HEK293 cells with PTX for 12 h did not affect cell viability (Additional file 1: Figure S1). Interestingly, an overnight (12 h) pretreatment with PTX resulted in a significant inhibition of ERK1/2 phosphorylation at late time points (≥20 min), whereas the early stage of ERK1/2 increase (at 5 min) was not blocked by PTX incubation (Fig. 3d). This suggests that FFA1 activates the ERK1/2 signaling pathway not only through a Gq-dependent pathway, but a Gi-dependent pathway. Take together, there is no involvement of Gβγ and arrestins in the FFA1 downstream ERK1/2 signal pathway, but Gq and Gi are both involved in regulating late-stage ERK1/2 activation.

## Discussion

FFA1 has been generally accepted to play an important role in various physiological functions. It is recognized as a potential target for the treatment of diabetes, bone remodeling, inflammation and neurogenesis [9, 10, 28]. Signals from almost all GPCRs, including FFA1, activate the ERK signaling cascades, which are associated with variety of cellular processes, including proliferation, migration, survival and apoptosis. Several studies have revealed that in breast cancer cell lines, the ERK1/2 and PI3K pathways are involved in proliferation; in part via FFA1 [29]. In addition, numerous observations suggested FFA1/ERK1/2 activation exhibited anti-apoptotic function [30], supported neuronal survival [10] and memory, induced osteocyte apoptosis [31] and had anti-inflammatory effects such as granule release and gene expression in neutrophils [32]. As a consequence, there is significant interest in understanding ERK1/2 signaling. However, details on the specific signaling pathway and molecular mechanism linking FFA1 to ERK1/2 activation in HEK293 cells remained unclear. We therefore used HEK293 cells that were stably transfected with human FFA1 receptors to characterize FFA1-induced ERK1/2 activation.

HEK293 is a commonly employed cell line used for the molecular and functional characterization of the GPCR signal transduction mechanism. There is growing evidence indicating that the FFA1 downstream ERK1/2 signal pathway participates in the regulation of various physiological functions [10, 30–32]. For better understanding of the specific mechanism of FFA1-mediated ERK1/2 activation, we examined the time course of ERK1/2 phosphorylation. The HEK293 cell lines stably expressing FFA1 showed a time-dependent activation of ERK1/2, peaking at 10 min and returning to the basal level at 60 min.

Fasiglifam previously demonstrated that the activation of FFA1 amplifies glucose-stimulated insulin secretion (GSIS) via IP3-induced Ca^2+^ oscillations and DAG/PKC-independent Ca^2+^ oscillations pathway [24]. We wanted to confirm the role of FFA1 downstream signal pathways in regulating ERK1/2 phosphorylation.

To examine if the dominant pathway for FFA1 to induce ERK1/2 phosphorylation is through G protein coupling, we first explored the role of Gq in the activation of ERK1/2. Upon activation of LA, ERK1/2 activation was significantly attenuated by treatment with the Gq inhibitor UBO-QIC. Our data demonstrated that the FFA1-mediated ERK1/2 activation was inhibited by the PKC inhibitor Go6983, suggesting that the PKC pathway is involved in ERK1/2 activation.

Pancreatic β-cells exhibit Ca^2+^ oscillations of variable frequency and amplitude, triggering pulsatile insulin secretion [33]. Therefore, experiments were conducted to elucidate whether Ca^2+^ mobilization of FFA1 has a role in ERK1/2 phosphorylation. Our results showed that FFA1-induced ERK1/2 activation was potently inhibited by the Ca^2+^ chelator EGTA and endoplasmic reticulum Ca^2+^ ATPase inhibitor thapsigargin at a late time point (≥ 20 min), indicating that Gq or another G protein caused calcium mobilization at a late time point through intracellular calcium release.

An interesting and important observation from our study is that the phosphoinositide-specific phospholipase C (PI-PLC) inhibitor edelfosine significantly blocked GRP40-induced ERK1/2 phosphorylation. PI-PLC is involved in the regulation of a large variety of cellular processes both in the plasma membrane and in the nucleus [34]. Although activation of PI-PLC was evidenced by an increase in the levels of two second messengers, IP3 and DAG, the nuclear inositide metabolism is regulated independently from that occurring elsewhere in the cell. Additional investigations will be necessary to clarify whether nuclear PI-PLC is involved in the FFA1 downstream signal pathway.

These data show that FFA1 mediates ERK1/2 phosphorylation through dual mechanisms, in which Gq/PI-PLC activate the IP3/Ca^2+^ and DAG/PKC signal pathways.

Further experiments were performed to examine whether or not EGFR tyrosine kinase, which is another mechanism in ERK1/2 signaling by GPCRs [35], is involved in FFA1. Our results demonstrate that in HEK293 cells, the EGF receptor-selective inhibitor AG1478 and the MMP inhibitor GM6001 do not impair ERK1/2 activation by FFA1 agonists. The Src family non-receptor tyrosine kinases have been proposed as early intermediates in the pathway toward inducing EGF receptor transactivation [36].

In our experiment, the Src-family tyrosine kinase inhibitor PP2 displayed no inhibition upon ERK1/2 activation by n-6-free fatty acid (LA). These results suggest that the transactivation of growth factor receptors does not participate in LA-induced ERK1/2 phosphorylation in HEK293 cells.

Previous studies revealed that as well as activating Gq, upon the administration of agonists, the Gi subunit can also be combined with FFA1, leading to a change in cell features [21, 22]. It has been reported that Gi- or Gq-coupled GPCRs activate calcium or ERK1/2 through the Gβγ subunit being released [37, 38]. Numerous distinct mechanisms allow activated GPCRs to signal through the ERK1/2 cascade. However, overexpression of the Gβγ scavenger proteins βARK-CT or Gα-transducin were found to have no effect on ERK1/2 phosphorylation.

Considerable evidence has accumulated implicating arrestins as signal transducers in the mediation of the ERK1/2 cascade [27]. However, knocking down arrestin-2 and -3 expression levels with specific siRNAs showed no effect on FFA1-induced ERK1/2 activation in HEK293 cells. This result suggests that arrestins are unlikely to play a major role in FFA1-mediated ERK1/2 activation.

Interestingly, overnight pretreatment of FFA1 stably expressing HEK293 has been found to significantly reduce ERK1/2 phosphorylation at late time points (≥ 20 min), especially when compared with the degree of increase in ERK1/2 at 5 min. It is conceivable that, upon stimulation by an agonist, FFA1 activates Gq proteins, leading to the activation of PI-PLC. This in turn results in the activation of the IP3/Ca^2+^ and DAG/PKC signaling pathways through activation of ERK1/2. After 20 min of stimulation by the agonist, the Gi involved in ERK1/2 phosphorylation, which may activate PI-PLC, leads to extracellular calcium influx. This notion is partially consistent with other reports demonstrating Gi/Gq-induced synergism in the regulation of intracellular Ca^2+^ mobilization and ERK1/2 phosphorylation [39, 40]. However, the Gβγ subunits are not involved in this synergism in the regulation of ERK1/2 activation. Additional investigations will be necessary to clarify how Gi is involved in FFA1-mediated ERK1/2 activation and its physiological function.

## Conclusion

This study provides a detailed delineation of the LA-mediated activation of ERK1/2 in FFA1-HEK293 cells. We propose that upon stimulation of LA, activated FFA1 causes the Gαq/11 protein to activate PI-PLC, causing the IP3/Ca^2+^ and DAG/PKC pathways to couple to ERK1/2 phosphorylation, and that there is no MMP/EGFR transactivation pathway involved in ERK1/2 activation. Further experiments confirmed that no arrestins, even Gβγ, are involved in FFA1-induced ERK1/2 activation. Surprisingly, we present evidence that Gi subunits play a critical role in ERK1/2 phosphorylation at late time points (≥ 20 min). These observations may provide new insights into the physiological functions modulated by FFA1-mediated activation of ERK1/2.

## Additional file

Additional file 1: Figure S1. Forskolin did not mimic the effect of PTX. A. Serum-starved FFA1-HEK293 cells were pretreated with DMSO or Forskolin (10μM) for 1h, and the cells were then stimulated with 10μM LA for the indicated time. ERK1/2 phosphorylation was assessed by Western blot as described in the Experimental Procedures and corresponding immunoblots were quantified by Bio-Rad Quantity One Imaging system. B. FFA1-HEK293 and HEK293 cells were exposed to PTX(100ng/ml) for indicated time, and than cell viabilities were evaluated by CCK8 assay at OD450nm. Error bars represent the SEM for three replicates. The data shown are representative of at least three replicate independent experiments. Data were analyzed using Student’s t-test (* p<0.001). (DOC 2835 kb)

## Abbreviations

DAG: Diacylglycerol
EGFR: Epidermal growth factor receptor
ERK1/2: Extracellular signal-regulated kinase 1 and 2
IP3: Inostiol 1,4,5-triphosphate
LA: Linoleic acid
MMP: Matrix metalloproteinase
PI-PLC: Phosphatidylinositol-specific phospholipase C
PKA: Protein kinase A
PKC: Protein kinase C
PTX: Pertussis toxin

## References

[1] Sawzdargo M (1997). A cluster of four novel human G protein-coupled receptor genes occurring in close proximity to CD22 gene on chromosome 19q13.1. Biochem Biophys Res Commun.

[2] Itoh Y (2003). Free fatty acids regulate insulin secretion from pancreatic beta cells through GPR40. Nature.

[3] Shapiro H, Shachar S, Sekler I, Hershfinkel M, Walker MD (2005). Role of GPR40 in fatty acid action on the beta cell line INS-1E. Biochem Biophys Res Commun.

[4] Wu J (2012). Inhibition of GPR40 protects MIN6 beta cells from palmitate-induced ER stress and apoptosis. J Cell Biochem.

[5] Nakamoto K, Tokuyama S (2015). [the possibility of a novel pain control system through brain long chain fatty acid receptor GPR40/FFAR1]. *Nihon yakurigaku zasshi*. Folia Pharmacologica Japonica.

[6] Nagasumi K (2009). Overexpression of GPR40 in pancreatic beta-cells augments glucose-stimulated insulin secretion and improves glucose tolerance in normal and diabetic mice. Diabetes.

[7] Steneberg P, Rubins N, Bartoov-Shifman R, Walker MD, Edlund H (2005). The FFA receptor GPR40 links hyperinsulinemia, hepatic steatosis, and impaired glucose homeostasis in mouse. Cell Metab.

[8] Wauquier F (2013). The free fatty acid receptor G protein-coupled receptor 40 (GPR40) protects from bone loss through inhibition of osteoclast differentiation. J Biol Chem.

[9] Fujita T (2011). A GPR40 agonist GW9508 suppresses CCL5, CCL17, and CXCL10 induction in keratinocytes and attenuates cutaneous immune inflammation. J Invest Dermatol.

[10] Zamarbide M (2014). GPR40 activation leads to CREB and ERK phosphorylation in primary cultures of neurons from the mouse CNS and in human neuroblastoma cells. Hippocampus.

[11] Lorenz K, Schmitt JP, Schmitteckert EM, Lohse MJ (2009). A new type of ERK1/2 autophosphorylation causes cardiac hypertrophy. Nat Med.

[12] Ahn S, Kim J, Hara MR, Ren XR, Lefkowitz RJ (2009). {beta}-Arrestin-2 mediates anti-apoptotic signaling through regulation of BAD Phosphorylation. J Biol Chem.

[13] Yoon S, Seger R (2006). The extracellular signal-regulated kinase: multiple substrates regulate diverse cellular functions. Growth Factors.

[14] Fujiwara K, Maekawa F, Yada T (2005). Oleic acid interacts with GPR40 to induce Ca2+ signaling in rat islet beta-cells: mediation by PLC and L-type Ca2+ channel and link to insulin release. *American journal of physiology*. Endocrinol Metab.

[15] Morgan NG, Dhayal S (2009). G-protein coupled receptors mediating long chain fatty acid signalling in the pancreatic beta-cell. Biochem Pharmacol.

[16] Soto-Guzman A, Robledo T, Lopez-Perez M, Salazar EP (2008). Oleic acid induces ERK1/2 activation and AP-1 DNA binding activity through a mechanism involving Src kinase and EGFR transactivation in breast cancer cells. Mol Cell Endocrinol.

[17] Hopkins MM, Zhang Z, Liu Z, Meier KE. Eicosopentaneoic acid and other free fatty acid receptor agonists inhibit Lysophosphatidic acid- and epidermal growth factor-induced proliferation of human breast cancer cells. J Clin Med. 2016;5(2):16–30 10.3390/jcm5020016 PMC4773772 26821052

[18] Kim MH, Kim MO, Kim YH, Kim JS, Han HJ (2009). Linoleic acid induces mouse embryonic stem cell proliferation via Ca2+/PKC, PI3K/Akt, and MAPKs. Cell Physiol Biochem.

[19] Zhang Y (2007). The role of G protein-coupled receptor 40 in lipoapoptosis in mouse beta-cell line NIT-1. J Mol Endocrinol.

[20] Hubbard KB, Hepler JR (2006). Cell signalling diversity of the Gqalpha family of heterotrimeric G proteins. Cell Signal.

[21] Schroder R (2011). Applying label-free dynamic mass redistribution technology to frame signaling of G protein-coupled receptors noninvasively in living cells. Nat Protoc.

[22] Feng DD (2006). Reduction in voltage-gated K+ currents in primary cultured rat pancreatic beta-cells by linoleic acids. Endocrinology.

[23] Qian J (2014). Differential requirements of arrestin-3 and clathrin for ligand-dependent and -independent internalization of human G protein-coupled receptor 40. Cell Signal.

[24] Sakuma K (2016). Fasiglifam (TAK-875) has dual potentiating mechanisms via Galphaq-GPR40/FFAR1 signaling branches on glucose-dependent insulin secretion. Pharmacol Rese Perspect.

[25] Amorino GP, Deeble PD, Parsons SJ (2007). Neurotensin stimulates mitogenesis of prostate cancer cells through a novel c-Src/Stat5b pathway. Oncogene.

[26] Dorn GW, Oswald KJ, McCluskey TS, Kuhel DG, Liggett SB (1997). Alpha 2A-adrenergic receptor stimulated calcium release is transduced by Gi-associated G(beta gamma)-mediated activation of phospholipase C. Biochemistry.

[27] Lefkowitz RJ, Shenoy SK (2005). Transduction of receptor signals by beta-arrestins. Science.

[28] Burant CF (2013). Activation of GPR40 as a therapeutic target for the treatment of type 2 diabetes. Diabetes Care.

[29] Hardy S, St-Onge GG, Joly E, Langelier Y, Prentki M (2005). Oleate promotes the proliferation of breast cancer cells via the G protein-coupled receptor GPR40. J Biol Chem.

[30] Panse M (2015). Activation of extracellular signal-regulated protein kinases 1 and 2 (ERK1/2) by free fatty acid receptor 1 (FFAR1/GPR40) protects from palmitate-induced beta cell death, but plays no role in insulin secretion. Cell Physiol Biochem.

[31] Mieczkowska A, Basle MF, Chappard D, Mabilleau G (2012). Thiazolidinediones induce osteocyte apoptosis by a G protein-coupled receptor 40-dependent mechanism. J Biol Chem.

[32] Mena SJ (2016). Differential free fatty acid receptor-1 (FFAR1/GPR40) signalling is associated with gene expression or gelatinase granule release in bovine neutrophils. Innate immunity.

[33] Nunemaker CS (2006). Glucose modulates [Ca2+]i oscillations in pancreatic islets via ionic and glycolytic mechanisms. Biophys J.

[34] Faenza I, Fiume R, Piazzi M, Colantoni A, Cocco L (2013). Nuclear inositide specific phospholipase C signalling - interactions and activity. FEBS J.

[35] Rozengurt E (2007). Mitogenic signaling pathways induced by G protein-coupled receptors. J Cell Physiol.

[36] Lin AL (2008). Distinct pathways of ERK activation by the muscarinic agonists pilocarpine and carbachol in a human salivary cell line. Am J Physiol Cell Physiol.

[37] Dickenson JM, Hill SJ (1998). Involvement of G-protein betagamma subunits in coupling the adenosine A1 receptor to phospholipase C in transfected CHO cells. Eur J Pharmacol.

[38] Gschwind A, Zwick E, Prenzel N, Leserer M, Ullrich A (2001). Cell communication networks: epidermal growth factor receptor transactivation as the paradigm for interreceptor signal transmission. Oncogene.

[39] Rebres RA (2011). Synergistic Ca2+ responses by G{alpha}i- and G{alpha}q-coupled G-protein-coupled receptors require a single PLC{beta} isoform that is sensitive to both G{beta}{gamma} and G{alpha}q. J Biol Chem.

[40] Chan AS, Yeung WW, Wong YH (2005). Integration of G protein signals by extracellular signal-regulated protein kinases in SK-N-MC neuroepithelioma cells. J Neurochem.

