# Supplementary material for: Agonist-induced activation of human FFA1 receptor signals to extracellular signal-regulated kinase 1 and 2 through Gq- and Gi-coupled signaling cascades
Source: Cell Mol Biol Lett. 2017 Jul 21;22:13. doi: 10.1186/s11658-017-0043-3 (PMC5522598; doi:10.1186/s11658-017-0043-3)
Supplement: Additional file 1: Figure S1. — Forskolin did not mimic the effect of PTX. A. Serum-starved FFA1-HEK293 cells were pretreated with DMSO or Forskolin (10μM) for 1h, and the cells were then stimulated with 10μM LA for the indicated time. ERK1/2 phosphorylation was assessed by Western blot as described in the Experimental Procedures and corresponding immunoblots were quantified by Bio-Rad Quantity One Imaging system. B. FFA1-HEK293 and HEK293 cells were exposed to PTX(100ng/ml) for indicated time, and than cell viabilities were evaluated by CCK8 assay at OD450nm. Error bars represent the SEM for three replicates. The data shown are representative of at least three replicate independent experiments. Data were analyzed using Student’s t-test (* p<0.001). (DOC 2835 kb) [file 11658_2017_43_MOESM1_ESM.doc]

Figure S1


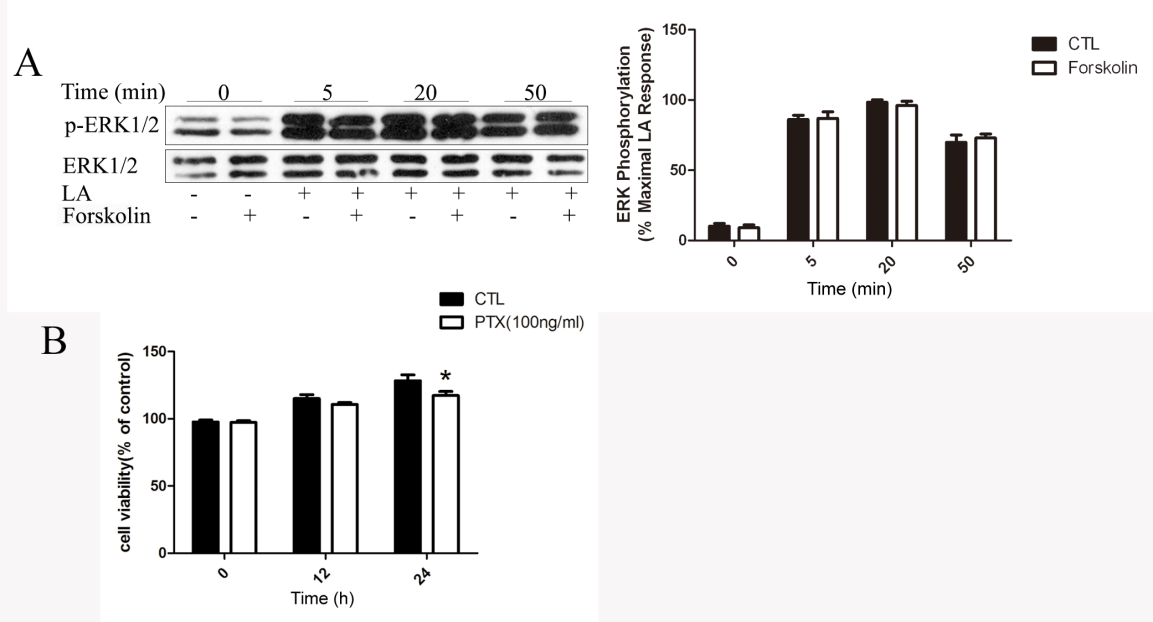


Figure S1: Forskolin did not mimic the effect of PTX. A.Serum-starved FFA1-HEK293 cells were pretreated with DMSO or Forskolin (10µM) for 1h, and the cells were then stimulated with 10µM LA for the indicated time. ERK1/2 phosphorylation was assessed by Western blot as described in the Experimental Procedures and corresponding immunoblots were quantified by Bio-Rad Quantity One Imaging system. B. FFA1-HEK293 and HEK293 cells were exposed to PTX(100ng/ml) for indicated time, and than cell viabilities were evaluated by CCK8 assay at OD450nm. Error bars represent the SEM for three replicates. The data shown are representative of at least three replicate independent experiments. Data were analyzed using Student’s t-test(* p<0.001).
